# Prevalence of menstrual disturbances and associated factors among female medical students in Southeast Asia: a systematic review and meta-analysis

**DOI:** 10.1186/s12905-026-04642-5

**Published:** 2026-07-03

**Authors:** Nguyen Dinh Ky, Nguyen Dang Linh Chi, Pham The Lam, Nguyen Ngoc Minh, Tran Trung Kien, Nguyen Si Hieu, Le Viet Hoang, Le Thi Ngan Ha, Nguyen Thi Huyen Anh

**Affiliations:** 1 Faculty of Medicine, Hanoi Medical University, Hanoi, Vietnam; 2 National Hospital of Obstetrics and Gynecology, Hanoi, Vietnam

**Keywords:** Menstrual disturbance, Dysmenorrhea, Irregular menstruation, Premenstrual syndrome, Female medical students, Southeast Asia, Prevalence, Associated factors, Academic stress, Absenteeism, PMD

## Abstract

**Background:**

Menstrual disturbances are common among female medical students and may affect academic functioning, well-being, and daily activities, especially in Southeast Asia. This systematic review and meta-analysis aimed to estimate the prevalence of menstrual disturbances and synthesize associated factors among female medical students in Southeast Asia.

**Methods:**

PubMed, Embase, Scopus, Google Scholar, manual searching, and citation tracking were searched for studies published from January 1, 2000 to May 6, 2026. Eligible studies were observational studies that reported the prevalence of menstrual disturbances and associated factors among female undergraduate medical students in Southeast Asia. Study quality was assessed using the Joanna Briggs Institute (JBI) checklist for analytical cross-sectional studies. Random-effects meta-analyses were conducted for outcomes with sufficient data, while associated factors were synthesized narratively.

**Results:**

17 studies involving 7,080 participants from Vietnam, Thailand, Indonesia, and Malaysia were included. Dysmenorrhea was the most frequently reported outcome, with a pooled prevalence of 78.11% (95% CI: 67.07%-86.21%; I² = 97.9%). The pooled prevalence was 30.10% for irregular menstruation (95% CI: 18.54%-44.89%; I² = 98.0%), 39.60% for premenstrual syndrome/premenstrual dysphoric disorder (95% CI: 20.13%-63.05%; I² = 98.5%), and 22.58% for abnormal menstrual cycle duration (95% CI: 15.48%-31.72%; I² = 95.0%). Family history showed the most consistent positive association with menstrual disturbances, with aOR ranging from 1.47 to 3.76. Psychological distress, functional impairment, caffeine intake, limited rest, and low or irregular physical activity were also reported as potential correlates, although evidence was heterogeneous. However, extremely high heterogeneity (I² >95%) was observed across all pooled outcomes; these estimates should therefore be interpreted with caution.

**Conclusions:**

Menstrual disturbances are commonly reported among female medical students in Southeast Asia. However, the evidence is limited by cross-sectional designs, inconsistent outcome definitions, high heterogeneity, and frequent risk of bias. These findings should therefore be interpreted as exploratory rather than confirmatory.

**Trial Registration:**

PROSPERO CRD420251178169.

**Supplementary Information:**

The online version contains supplementary material available at https://doi.org/10.1186/s12905-026-04642-5.

## Background

Menstrual disturbances (MDs), including dysmenorrhea, menstrual irregularity, and premenstrual syndrome (PMS), are common menstrual health conditions affecting cycle regularity, bleeding patterns, and related physical and psychological symptoms [1, 2]. Globally, these conditions are highly prevalent among reproductive-aged women and are associated with impaired quality of life, functional limitations, and reduced daily productivity [3–5]. Among university students, MDs have also been linked to concentration difficulties, absenteeism, and lower academic performance, highlighting their relevance in educational settings [6, 7].

Female medical students may be particularly vulnerable to MD because of the distinct demands of medical training. Compared with students in many other health-related disciplines, undergraduate medical education typically involves longer academic programs, intensive theoretical coursework, frequent examinations, prolonged clinical rotations, and irregular schedules, all of which may contribute to stress, sleep disruption, and altered health behaviors-factors consistently associated with menstrual symptoms and cycle disturbances [2, 8, 9]. A recent systematic review among medical students reported pooled prevalence estimates of 51.3% for PMS and 72.7% for dysmenorrhea, with stress, physical inactivity, and high caffeine intake identified as common associated factors [1]. These findings suggest that medical students represent a distinct educational subgroup in whom menstrual health warrants focused investigation.

The epidemiology and determinants of MD may also vary by regional context. In Southeast Asia (SEA), menstrual health is shaped by diverse sociocultural norms, differing levels of healthcare access, and variation in educational and clinical training environments across countries. Most SEA countries are low- and middle-income countries (LMICs), where menstrual health literacy, access to reproductive health services, and help-seeking behaviors may be influenced by resource constraints and cultural perceptions surrounding menstruation [10]. Emerging evidence from SEA also indicates that menstrual disturbances are common among female medical students in the region, with studies from Malaysia, Vietnam, and Indonesia reporting substantial frequencies of dysmenorrhea, menstrual irregularities, and menstruation-related academic disruption, varying around 70% to 90% [11–13]. In parallel, high levels of psychological distress, including stress, anxiety, and burnout, have been consistently documented among medical students in SEA, suggesting that the regional training environment may interact with behavioral and psychosocial determinants of menstrual health in context-specific ways [14]. These findings indicate that prevalence estimates and associated factors reported in other regions may not be directly generalizable to SEA.

Although several systematic reviews have examined menstrual disturbances among medical students internationally, evidence from SEA remains limited and fragmented, with available studies largely reported at the single-country level and varying in scope and methodology. Consequently, the regional burden of MD and the factors associated with it among female medical students in SEA have not been systematically synthesized. This evidence gap limits contextual understanding of menstrual health in a population essential to the future healthcare workforce in the region.

Therefore, this systematic review and meta-analysis aim to address this gap by estimating the pooled prevalence of menstrual disturbances among female medical students in Southeast Asia, with additional focus on factors associated with menstrual disturbances and contextual factors relevant to menstrual health within the regional medical training environment.

## Methods

Our study was conducted in strict accordance with the PRISMA 2020 guidelines. A detailed description of how each PRISMA 2020 reporting item was addressed is provided in Additional File 12.

### Protocol and registration

The systematic review was registered prospectively in PROSPERO with the registration number CRD420251178169 and registration date 28 October, 2025.

Several modifications were made during the conduct of the review compared with the original protocol. First, the search end date was extended from September 30, 2025 to May 06, 2026, because additional relevant studies were published during the revision period and including them strengthened the evidence base. Second, the eligible outcomes were refined to focus on operationally measurable menstrual disturbance subtypes, and the population criterion was clarified to require female undergraduate medical students, improving conceptual consistency and reducing clinical heterogeneity. No language restrictions were applied in either the original or revised protocol. These deviations were not pre-registered as amendments in PROSPERO because they were clarifications rather than substantive changes to the research question or analytic approach; however, they are fully disclosed here and in Additional file 14, which provides a side-by-side comparison of the original protocol criteria and the revised criteria with justification for each modification.

### Outcome definition

For this review, menstrual disturbances were operationally defined as self-reported menstrual health conditions involving abnormal menstrual symptoms or cycle characteristics, including dysmenorrhea, menstrual irregularity, premenstrual syndrome (PMS), and premenstrual dysphoric disorder (PMDD). These outcomes were analyzed as distinct symptom-specific conditions, depending on how they were reported in the primary studies.

### Search strategy

A comprehensive search of studies was conducted through the following databases: Pubmed, Embase, and Scopus. The timeframe was restricted from January 1, 2000, to May 06, 2026. The search terms consisted of Medical Subject Headings (MeSH) terms and keywords related to three core concepts: menstrual disturbances, the target population (female medical students), and the geographical context (Southeast Asia). Broader search terms encompassing associated factors were retained to ensure comprehensive capture of studies reporting both prevalence and correlates, consistent with the dual objectives of this review. To enhance search comprehensiveness, the first 200 relevance-ranked records on Google Scholar were additionally screened. The complete database-specific search strategies are provided in Additional file 1.

### Inclusion and exclusion criteria

Studies were eligible for inclusion if they met all of the following criteria: [1] the study population included female undergraduate medical students in Southeast Asia, or a mixed student population from which data for female undergraduate medical students could be extracted separately; [2] the study reported quantitative data on the prevalence of menstrual disturbances, either overall or by specific subtypes, and/or quantitative data on factors associated with menstrual disturbances; [3] the menstrual disturbance outcomes included at least one of the following: dysmenorrhea, irregular menstruation, premenstrual syndrome or premenstrual dysphoric disorder, abnormal menstrual cycle duration, heavy menstrual bleeding, or overall menstrual disorders as defined by the original study; [4] the study used an observational design, including cross-sectional, cohort, or case-control designs; [5] the study provided sufficient extractable data, such as numerators and denominators, prevalence estimates, effect estimates, confidence intervals, p-values, or data from which these could be derived; and [6] the full text was published or made publicly available between January 1, 2000 and May 6, 2026. No language restrictions were applied.

Reports were excluded if they were duplicate publications, used overlapping datasets without providing additional eligible data, or were published only as conference abstracts without sufficient full-text information for extraction. Reviews, systematic reviews, meta-analyses, editorials, commentaries, letters without original data, protocols, case reports, case series, and interventional studies were also excluded. For mixed-population studies, reports were excluded when data for female undergraduate medical students could not be separated from other student groups, health-profession groups, or general populations. Reports with insufficient quantitative data for extraction or calculation were excluded after full-text assessment.

### Study selection

**Fig. 1:**
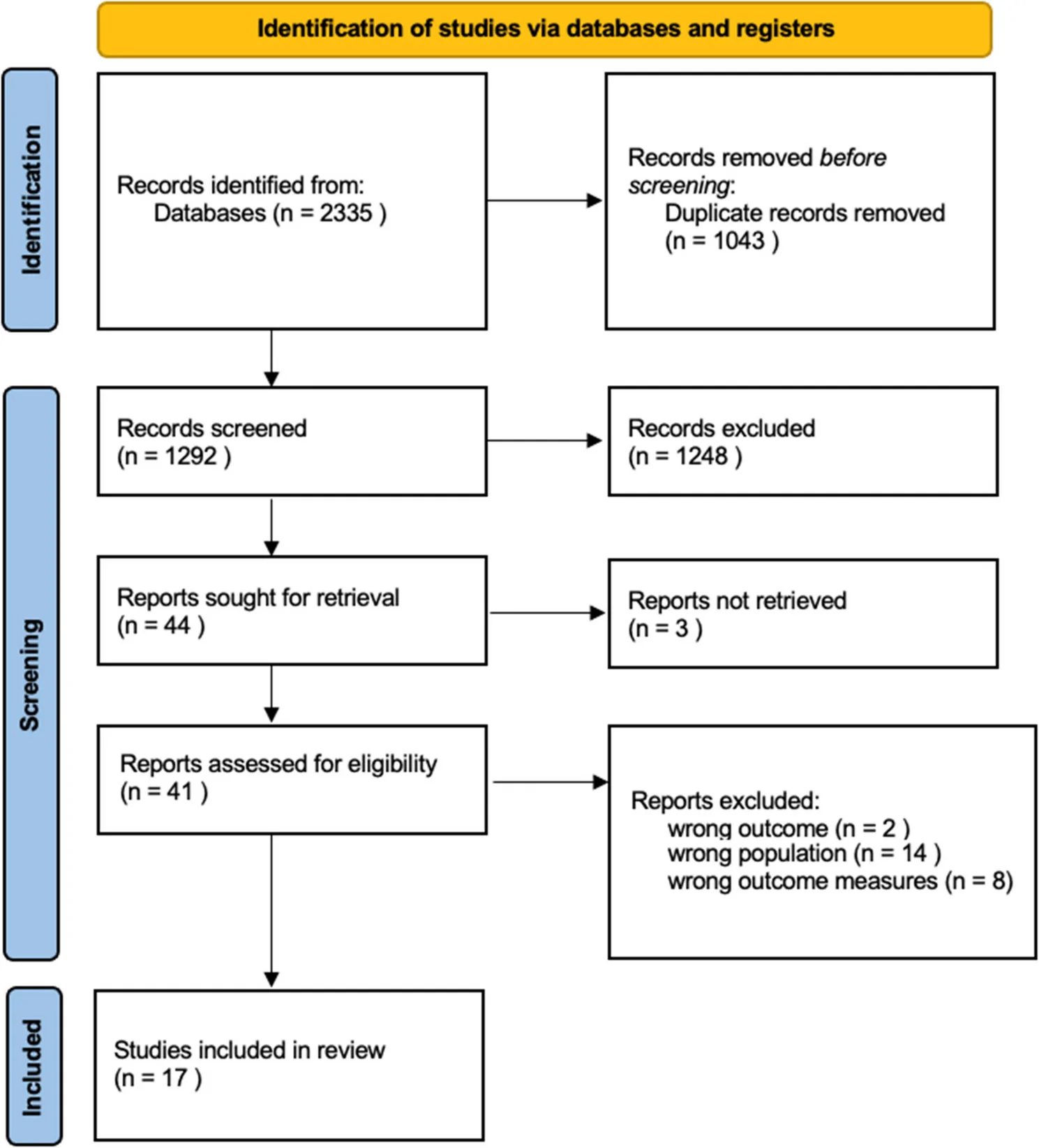
PRISMA flow diagram

The study selection followed the PRISMA guidelines [15]. First, all the identified studies were sent to the manager software Rayyan, and duplicates were deleted. Two reviewers independently performed the first selection and review of the remaining articles by reviewing titles and abstracts, after which irrelevant articles were removed on the basis of the inclusion and exclusion criteria. In the next step, to confirm their eligibility, the full texts of the remaining articles were evaluated with the same criteria, and any irrelevant articles were removed. Differences between the two reviewers were settled by the third reviewer. The study selection and screening processes are presented in the PRISMA flow diagram (Fig. 1).

### Data extraction and quality assessment

Data extraction was performed independently by two reviewers through a standardized data extraction form that was specifically designed for this review and pilot tested on a subset of the included studies. The extracted information included study characteristics, participant characteristics, reported symptoms and measurement methods of menstrual disorders, reported prevalence estimates, and associated factors. Adjusted effect estimates were preferentially extracted when reported. If adjusted estimates were not reported, crude odds ratios (ORs), prevalence ratios (PRs), or prevalence odds ratios (PORs) were extracted. Corresponding 95% confidence intervals and *p*-values were recorded whenever available. The complete data extraction from all included studies was provided in Additional File 13. As all included studies were cross-sectional, the quality assessment was performed through the JBI Analytical Cross-Sectional Studies checklist [16]. The appraisal was organized into the following domains: clarity of inclusion criteria and participant description, validity/reliability of exposure and outcome measurement, identification and management of confounding factors, and appropriateness of statistical analysis. The JBI total scores (0–8) were categorized as high [6–8], moderate ([4, 5]), or low (0–3). The certainty of evidence for each major prevalence outcome was assessed using the GRADE approach [17]. 

### Data analysis

Studies were grouped for each synthesis based on the specific menstrual outcome reported. We tabulated study characteristics and outcome types, and only studies reporting extractable prevalence data for a given outcome were included in the meta-analysis. Studies with insufficient quantitative data were synthesized narratively. The data were extracted from a Microsoft Excel data sheet and imported into R version 4.6.0. The heterogeneity was assessed using Cochran’s Q statistic, quantified using I^2^, and presented in a forest plot. A random-effects model was prespecified and applied to account for both within-study and between-study variability in participant characteristics, outcome definitions, and measurement methods. Statistical heterogeneity was assessed using Cochran’s Q test and quantified using I², with *p* < 0.10 for Q or I² > 50% interpreted as indicating substantial heterogeneity. Prevalence estimates were pooled with corresponding 95% confidence intervals, and appropriate transformations were applied to stabilize variances when necessary. If there were variations across the primary studies, subgroup analyses by geographical characteristics were conducted.

For associated factors, we did not perform a meta-analysis because studies were substantially heterogeneous in effect measures, model specifications, and adjustment strategies. Instead, we used a structured narrative approach that summarised effect estimates and counted votes based on the direction of the effect. In interpreting associated factors, greater weight was given to studies that reported adjusted estimates (aORs) and achieved moderate-to-high quality ratings according to the JBI checklist. The heterogeneity of associated factors was explored informally by comparing methodological characteristics and measurement instruments across studies.

Publication bias was assessed for outcomes with at least three included studies using funnel plot inspection. Egger’s regression test was additionally performed when at least 10 studies were available, with *p*-value < 0.10 considered suggestive of potential small-study effects. Sensitivity analyses were conducted using leave-one-out methods and by excluding studies judged to have a high risk of bias to assess the robustness and stability of pooled estimates.

## Results

### Study identification

The search identified 2,335 records, of which 1,043 duplicates were removed. After title and abstract screening, 44 reports were sought for retrieval; three were not retrieved. Among 41 full-text reports assessed for eligibility, 24 were excluded for predefined reasons, which are listed in Additional file 11. Finally, 17 studies were included in the qualitative synthesis. The study selection process is summarized in Fig. 1.

### Description of studies

Table 1 provides descriptive details of the included studies. The included studies were published between 2012 and 2025. The total sample size across all included studies was 7,080 participants, with individual study sample sizes ranging from 126 to 922 participants. Geographically, the studies were conducted across 4 SEA countries, including Vietnam, Thailand, Indonesia, and Malaysia.

**Table 1:** Description of the population and sample in the selected studies

Study publication year (first author, year)	Country	Participants	Academic year	Sample size	Sampling
Herbert Situmorang, 2024 [12]	Indonesia	Undergraduate medical students	Not mentioned	630	Snowball sampling
Fildzah Hasifa Taufiq, 2019 [18]	Indonesia	Undergraduate medical students	Not mentioned	503	Total sampling technique
Heethal Jaiprakash, 2016 [19]	Malaysia	Undergraduate medical students	Y1 - Y3	215	Non - probability convenient sampling method
Htoo Htoo Kyaw Soe, 2017 [11]	Malaysia	Undergraduate medical students	Not mentioned	292	Purposive sampling
Prasong Tanmahasamut, 2012 [20]	Thailand	Undergraduate medical students	Y2 - Y6	552	Not mentioned
Vy Dinh Trieu Ngo, 2023 [21]	Vietnam	Undergraduate medical students	Not mentioned	302	Convenience sampling
Nam Hoang Tran, 2025 [22]	Vietnam	Medical students extracted only	Not mentioned	884	Not mentioned
Le Xuan Hung, 2024 [23]	Vietnam	Undergraduate medical students	Y1 - Y6	231	Convenience sampling
Tran Thi Quynh Trang, 2023 [24]	Vietnam	Undergraduate medical students	Y1 - Y6	412	Convenience sampling
Khanh Hoang Pham, 2025 [25]	Vietnam	Undergraduate medical students	Y3	126	Not mentioned
Ho Ngoc Lan Nhi, 2024 [26]	Vietnam	Undergraduate students in Medicine, Pharmacy, Dentistry, Nursing and Traditional Medicine programs	Y1 - Y6	385	Simple random sampling
Le Nguyen Quynh Nhu, 2024 [27]	Vietnam	Undergraduate medical students	Not mentioned	360	Convenience sampling
Herbert Situmorang, 2025 [28]	Indonesia	Undergraduate medical students	Not mentioned	630	Snowball sampling
Do Thanh Tung, 2024 [29]	Vietnam	Undergraduate medical students	Y1 - Y6	312	Convenience sampling
Vu Dinh Nam, 2025 [30]	Vietnam	Undergraduate medical students	Not mentioned	152	Census
Do Tuan Dat, 2022 [13]	Vietnam	Undergraduate medical students	Not mentioned	922	Not mentioned
Sebastian Thevaraja Emmanuel Joseph, 2013 [31]	Malaysia	Undergraduate medical students	Y1 - Y2	172	Simple random sampling

“Not mentioned” indicates that the information was not reported in the original publication

Regarding outcomes, 3 studies reported the overall prevalence of menstrual disturbances, whereas the remaining studies focused on specific subtypes [18, 25, 29]. Dysmenorrhea was the most frequently reported outcome (9 studies) [11–13, 19, 20, 25, 26, 28, 30], followed by irregular menstruation (6 studies) [11, 22, 24, 25, 30, 31], premenstrual disorders, including PMS and PMDD (5 studies) [11, 22–24, 32], and AMCD (5 studies) [11, 12, 18, 28, 31]. 

When using the JBI checklist for quality assessment, the majority of studies were assessed as having low to moderate quality, with most meeting key criteria related to the inclusion criteria and the study population description. Notably, confounding factors have seldom been addressed, and many studies have provided limited information on the validity of exposure or outcome measurements. Furthermore, the use of appropriate statistical analyses was inconsistent, with several studies failing to report analytic strategies in sufficient detail (Additional file 2).

Using the GRADE approach, the certainty of evidence was rated as very low for all pooled prevalence outcomes. Although dysmenorrhea, irregular menstruation, PMS/PMDD, and AMCD were consistently reported across studies, the certainty of the pooled estimates was downgraded mainly because of serious risk of bias, serious inconsistency, and serious imprecision. The very high heterogeneity across outcomes, broad prediction intervals, inconsistent outcome definitions, and frequent use of non-probability sampling substantially reduced confidence in the pooled estimates. For associated factors, the certainty of evidence was also generally low because all included studies were cross-sectional, adjustment for confounding was inconsistent, and exposure and outcome measurements varied across studies. (Additional file 3)

### Synthesis of results

**Fig. 2:**
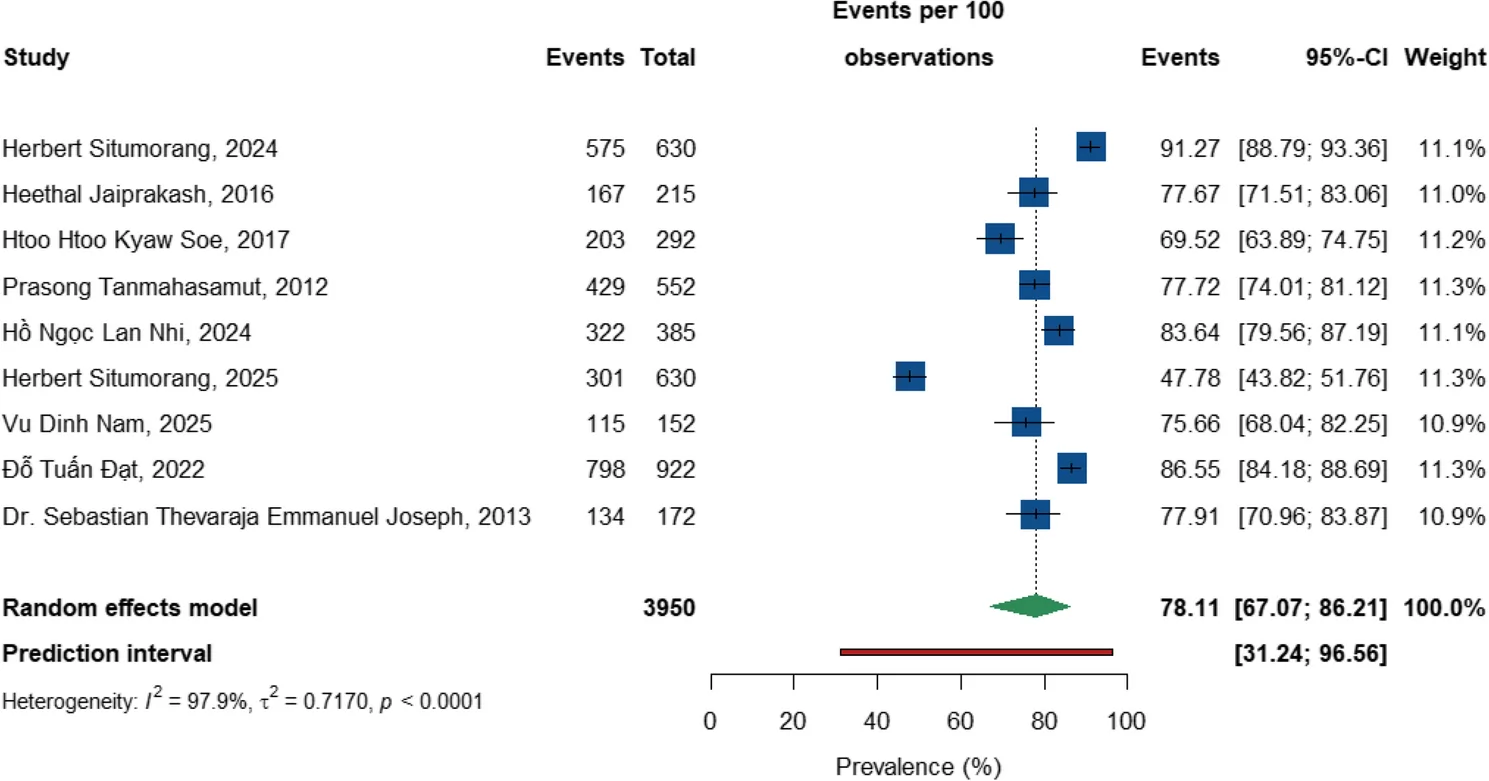
Forest plot of the pooled prevalence of dysmenorrhea

9 studies reported the prevalence of dysmenorrhea among medical students in Southeast Asia. The pooled prevalence was 78.11% (95% CI: 67.07%-86.21%). Across studies, the reported prevalence varied widely, ranging from 47.8% to 91.3%. There was considerable heterogeneity between studies (I² = 97.9%). (Fig. 2)

**Fig. 3:**
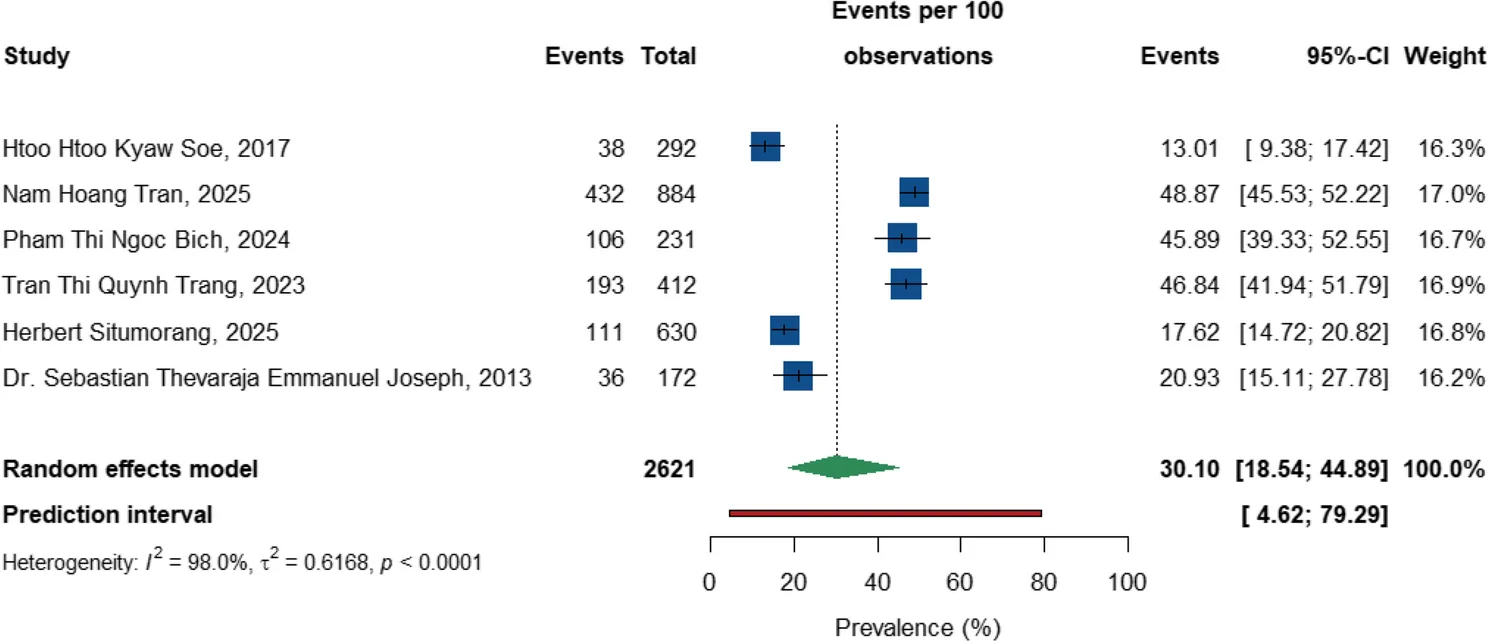
Forest plot of the pooled prevalence of irregular menstruation

6 studies evaluated irregular menstruation. The pooled prevalence was 30.10% (95% CI: 18.54%-44.89%), with a high degree of heterogeneity (I² = 98.0%). (Fig. 3)

**Fig. 4:**
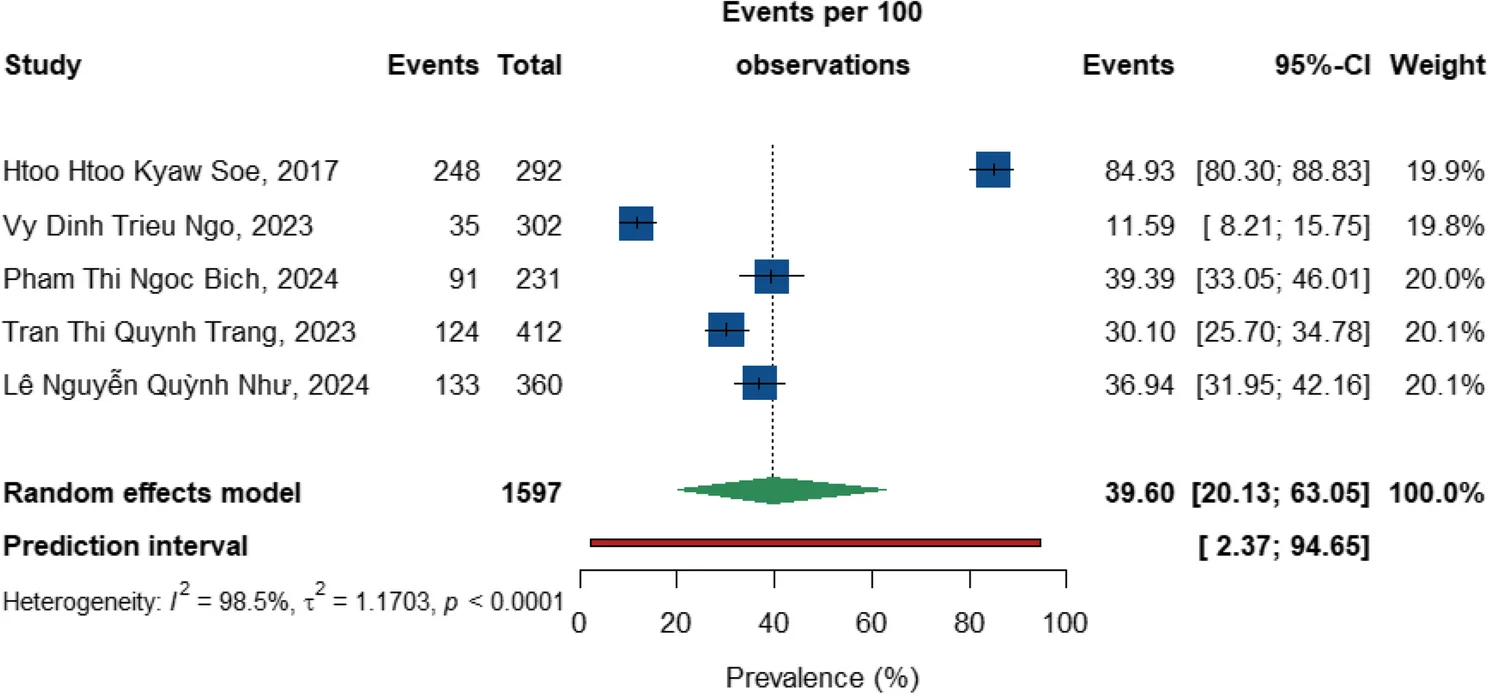
Forest plot of the pooled prevalence of premenstrual disorders

5 studies contributed data on premenstrual disorders, including PMS and PMDD. The pooled prevalence was 39.60% (95% CI: 20.13%-63.05%). Individual study estimates showed substantial variation, ranging from 11.6% to 84.9%. Heterogeneity remained high (I² = 98.5%). (Fig. 4)

**Fig. 5:**
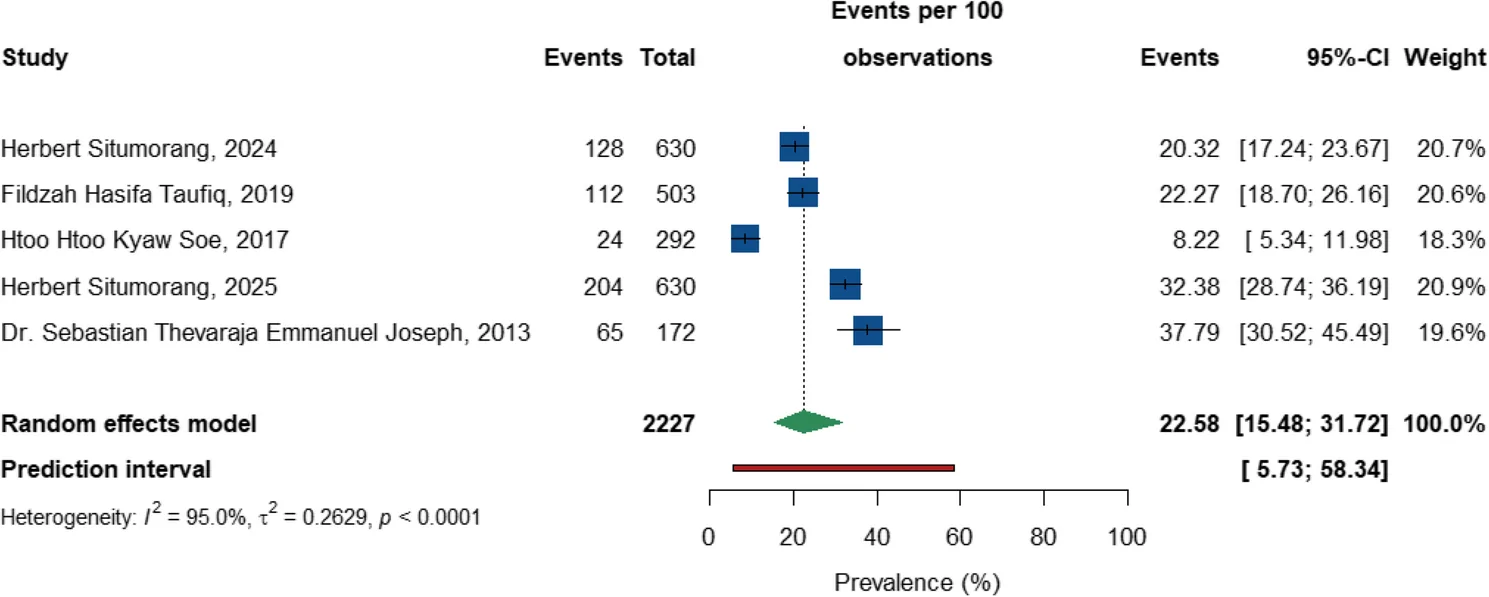
Forest plot of the pooled prevalence of abnormal menstrual cycle duration

Abnormal menstrual cycle duration (AMCD) was reported in 5 studies. The pooled prevalence was 22.58% (95% CI: 15.48%-31.72%), with the reported prevalence ranging from 8.2% to 37.8%. High between-study heterogeneity persisted (I² = 95.0%). (Fig. 5)

Three studies provided data for overall menstrual disorders. Taufiq et al. (2019) in Indonesia reported a prevalence of 22.3%, while Do Thanh Tung et al. (2024) and Khanh Hoang Pham et al. (2024) in Vietnam reported the prevalence of 55.5% and 39.7%, respectively [18, 25, 29]. 

Heavy menstrual bleeding (HMB) was reported in three studies conducted among medical students in Southeast Asia. In an Indonesian study by Situmorang et al. (2024), 122 of 630 participants reported HMB, corresponding to a prevalence of 19.4% [12]. In a Malaysian study by Kyaw Soe et al. (2017), 80 of 292 participants reported HMB, yielding a prevalence of 27.4% [11]. Another study in Malaysia by Joseph et al. (2013) reported HMB in 57% of the participants [31]. 

Only one study by Nam Hoang Tran et al. (2025) reported menstrual change with a prevalence of 49.8% [25]. 

Meta-analyses for menstrual disorders, HMB, and menstrual change were not performed due to the limited number of studies and methodological heterogeneity.

### Subgroup analysis

**Fig. 6:**
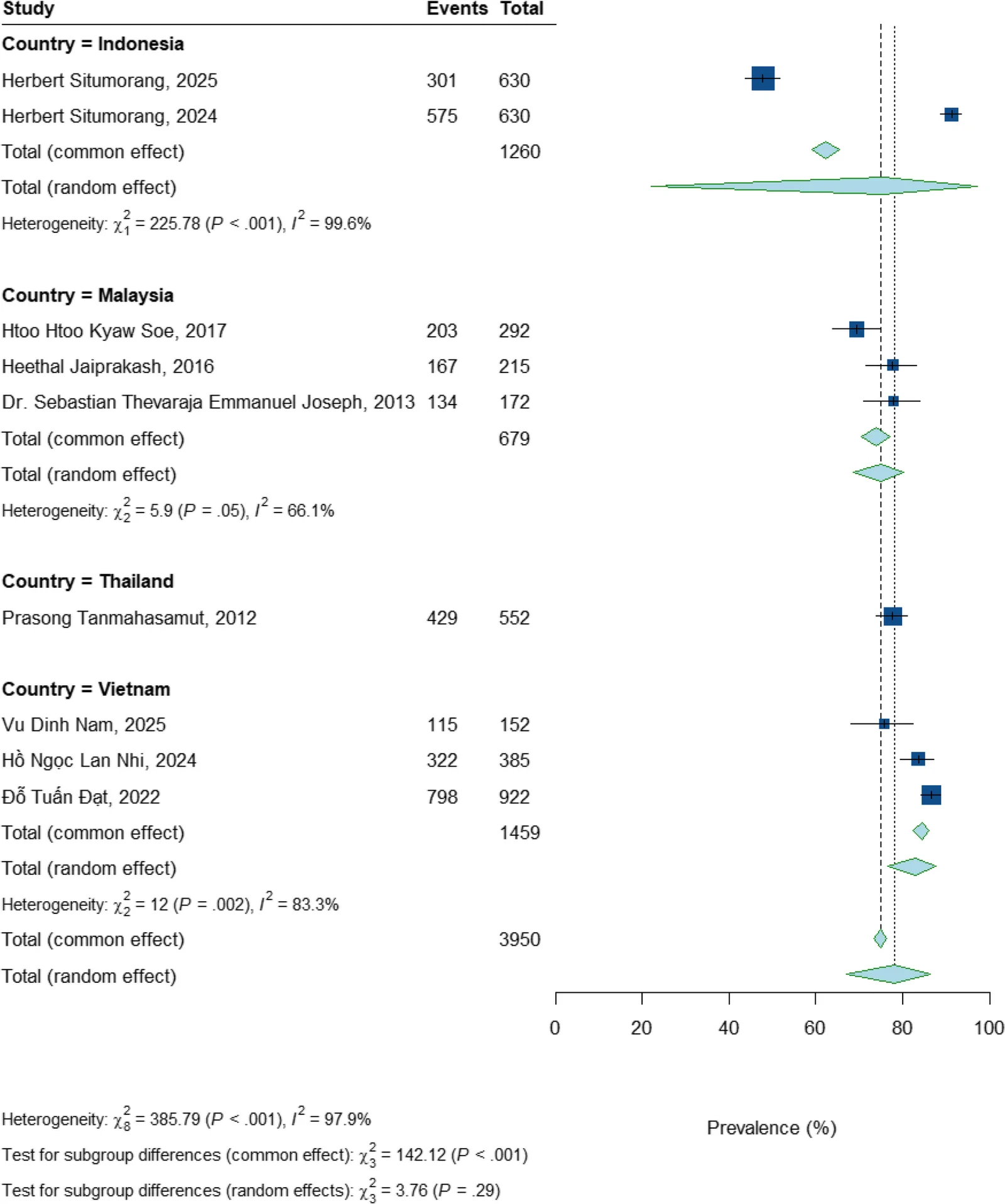
Subgroup analysis of pooled prevalence of dysmenorrhea by country

Subgroup analyses stratified by country were performed for dysmenorrhea, irregular menstruation, and AMCD. For dysmenorrhea, the result revealed statistically insignificant geographical differences (*p* = 0.29 for subgroup differences test using random-effects models). Moreover, despite stratification, high within-group heterogeneity persisted, particularly within the Indonesian subgroups (I^2^ = 99.6%, *p* < 0.001). (Fig. 6)

**Fig. 7:**
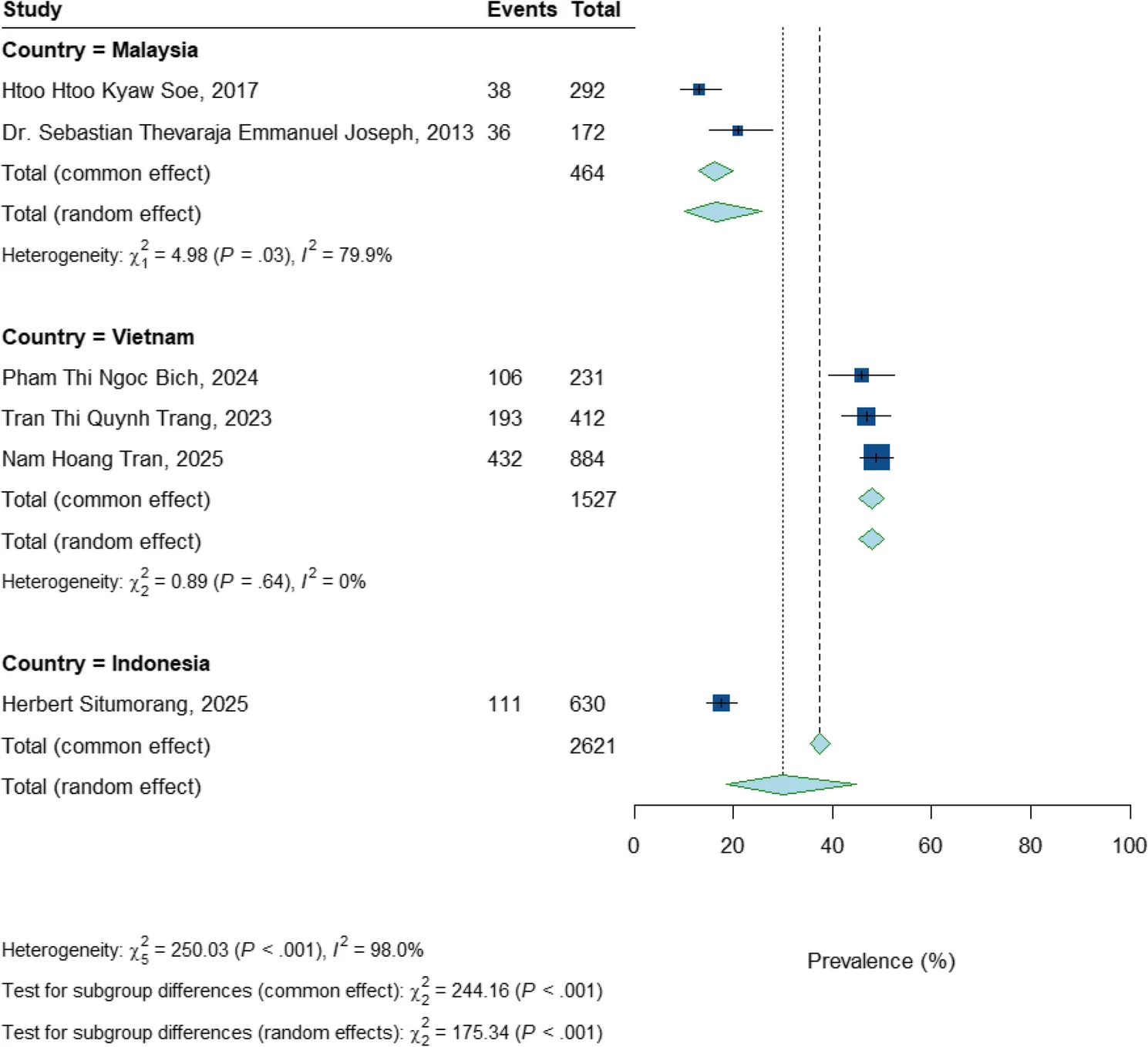
Subgroup analysis of pooled prevalence of irregular menstruation by country

In contrast, subgroup analysis for irregular menstruation revealed significant differences between countries (*p* < 0.001). Studies from Vietnam showed relatively consistent prevalence estimates with no statistically significant heterogeneity observed (I² = 0%, *p* = 0.64), whereas substantial heterogeneity was observed among Malaysian studies (I² = 79.9%, *p* = 0.03). (Fig. 7).

**Fig. 8:**
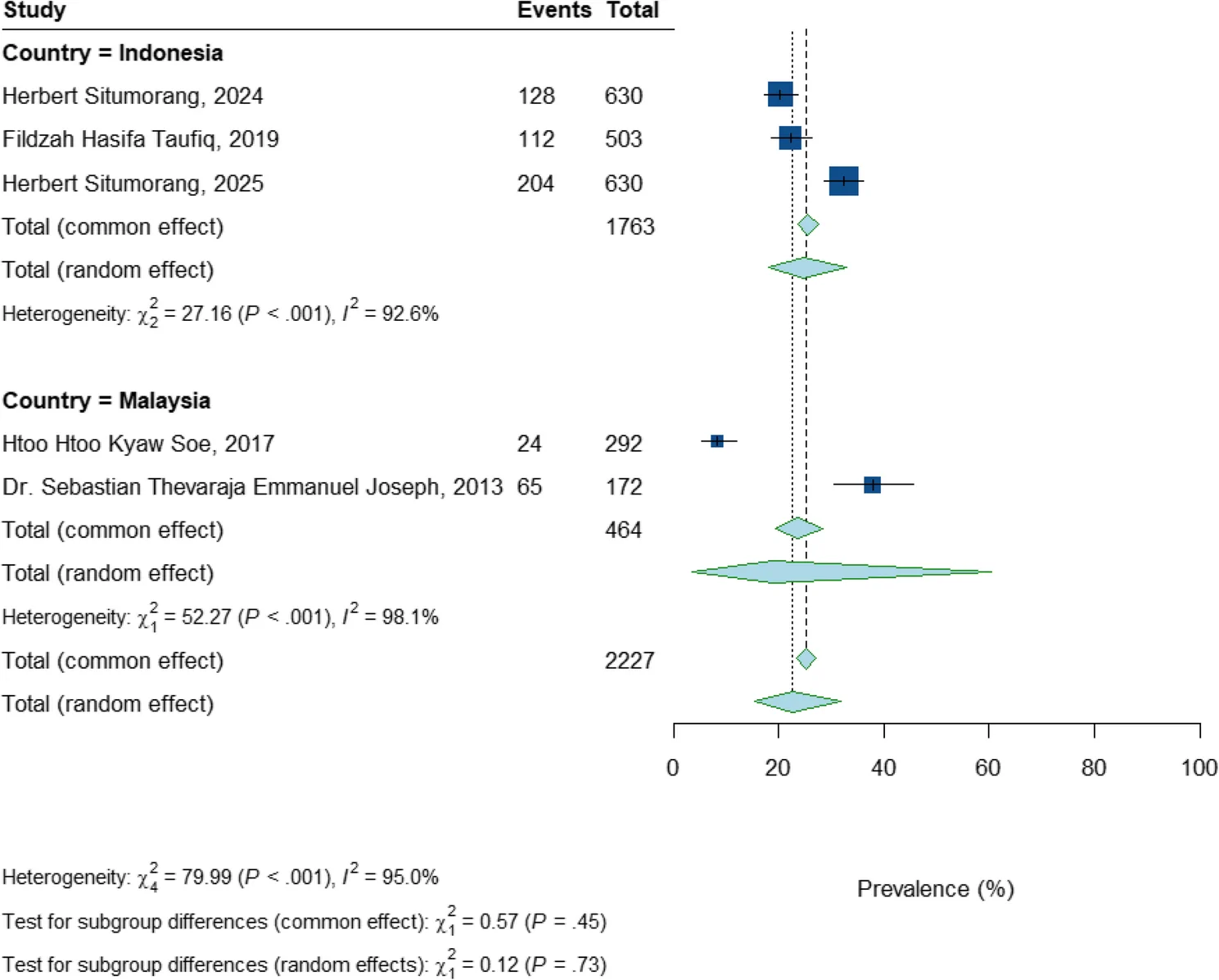
Subgroup analysis of pooled prevalence of abnormal menstrual cycle duration by country

For AMCD, no significant subgroup differences were observed between countries under the random-effects model (p = 0.73). However, heterogeneity remained considerable within both Indonesian (I² = 92.6%, *p* < 0.001) and Malaysian studies (I² = 98.1%, p < 0.001). (Fig. 8).

However, interpretation of these findings should be approached cautiously, given the limited number of studies within subgroups. 

Subgroup analyses for premenstrual disorders were not performed because too few studies were available within each country subgroup to permit meaningful comparisons.

### Sensitivity analysis

Sensitivity analyses using the leave-one-out method demonstrated that the pooled estimates for all outcomes were generally robust, with no individual study having a dominant influence on the overall results. Nevertheless, the 95% prediction intervals remained wide across all outcomes: 31.24%-96.56% for dysmenorrhea, 4.62%-79.29% for irregular menstruation, 2.37%-94.65% for premenstrual disorders, and 5.73%-58.34% for AMCD, reflecting substantial contextual variation and indicating that future studies conducted in comparable settings may yield heterogeneous prevalence estimates. Moreover, heterogeneity was persistently high after sequential exclusion of individual studies, with I² values consistently exceeding 92% for dysmenorrhea, 97% for irregular menstruation, 95% for premenstrual disorders, and 92% for AMCD. These findings suggest that the observed heterogeneity is not attributable to any single study but rather reflects underlying differences across study settings and methodologies. (Additional file 4)

Furthermore, exclusion of studies with a high risk of bias in the sensitivity analysis for dysmenorrhea yielded a pooled prevalence estimate of 78.09% (95% CI 62.05%-88.60%), comparable to that of the primary analysis (78.11%), while heterogeneity remained substantial (I² = 98.4%, *p* < 0.0001). Together, these findings support the robustness of the pooled estimates and suggest that the observed heterogeneity is unlikely to be driven solely by lower-quality studies. (Additional file 5) In contrast, sensitivity analyses excluding studies with a high risk of bias could not be performed for irregular menstruation, premenstrual disorders, and AMCD because the number of remaining eligible studies after exclusion was insufficient to support reliable meta-analytic estimation (k < 5).

### Publication bias

Publication bias was explored using contour-enhanced funnel plots for studies reporting dysmenorrhea (Additional file 6), irregular menstruation (Additional file 7), PMS/PMDD (Additional file 8), and AMCD (Additional file 9). Egger’s regression test was not performed due to the limited number of studies for each outcome (k < 10). Although visual inspection suggested some degree of asymmetry, interpretation is limited given the small number of studies and substantial heterogeneity across analyses (I² > 95%). Therefore, the funnel plots are presented for illustrative purposes only, and no definitive conclusions regarding publication bias can be drawn.

### Factors associated with menstrual disturbances

We did not perform a meta-analysis for associated factors because studies were substantially heterogeneous in effect measures, model specifications, and adjustment strategies. Therefore, a narrative synthesis was chosen as our approach. The estimates summarized below were taken from studies with JBI scores of 5 or above, whereas findings from all 17 studies are provided in Additional file 10. Associated factors are organized into 5 thematic domains corresponding to the subheadings below: family history; reproductive and medical history; psychological status and quality of life; demographic and academic characteristics; and dietary habits and lifestyle.

### Family history

Family history was the most consistent correlate. In vote counting, all studies reporting this factor showed a positive association with dysmenorrhea or menstrual disorders (5/5 studies reporting this factor; 5/17 studies overall) [12, 13, 19, 20, 29]. Family history was associated with primary dysmenorrhea occurrence and severity (aOR range 1.47–3.76), and with general dysmenorrhea in another study (OR 2.69, 95% CI 1.37–5.25) [12, 19]. 

### Reproductive and medical history

Menstrual characteristics also showed strong and largely consistent associations with adverse outcomes. Dysmenorrhea was positively associated with PMS in all studies reporting this relationship (2/2 studies), with a study reporting OR 14.6 (95% CI 2.9–73.2) [22]. Heavy menstrual bleeding was also associated with PMS, with aOR 25.7 (95% CI 2.5- 265.3); [22] however, the wide confidence interval for the adjusted estimate suggests imprecision. Negative Rh blood type was associated with PMS/PMDD (PR 4.43, 95% CI 1.95–10.08) [21]. 

### Psychological and quality of life

Stress was linked to dysmenorrhea severity (aOR 1.82, 95% CI 1.26–2.62), while depression was associated with both dysmenorrhea occurrence and severity (aOR range 2.07–2.16). Functional impacts were substantial, including concentration disruption (aOR range 7.24–12.92), activity disruption (aOR range 6.92–34.95), and absenteeism (aOR range 5.65–12.10) [28]. The larger estimate for activity disruption probably reflects differences in outcome definition, as one model assessed dysmenorrhea occurrence whereas the other assessed severity. Dysmenorrhea was also associated with lower adjusted physical and mental HRQoL scores (adjusted mean difference − 4.9 and − 3.4, respectively; both *p* < 0.001) [31]. 

### Demographic and academic factors

Demographic and academic factors were less consistent. Age and clinical academic level were generally not associated with menstrual outcomes in higher-priority studies, whereas Malay ethnicity showed elevated odds of dysmenorrhea (aOR 5.70, 95% CI 1.13–28.78) and Indonesian students of foreign descent had lower odds of primary dysmenorrhea severity (aOR 0.50, 95% CI 0.30–0.83) [12, 19]. 

### Dietary habits and lifestyle

Lifestyle-related findings were more heterogeneous. Frequent caffeine intake was associated with PMS/PMDD in the only high-priority study reporting this factor (PR 2.86, 95% CI 1.24–6.58). In contrast, alcohol consumption and moderate or vigorous physical activity were not clearly associated with PMS/PMDD, and exercise showed only a non-significant positive estimate for PMS (aOR 2.40, 95% CI 0.90–5.80) [21, 22]. 

## Discussion

### Principal findings

This systematic review and meta-analysis showed that menstrual disturbances are common among female medical students in Southeast Asia. Overall, dysmenorrhea was the most prevalent condition, with a pooled prevalence of 78.11%, followed by PMS/PMDD at 39.60%, irregular menstrual cycles at 30.10%, and AMCD at 22.58%. Despite these high pooled prevalence estimates, substantial between-study heterogeneity was observed across all outcomes. In subgroup analyses, no statistically significant differences were found between countries, and heterogeneity remained high within each country. One exception was observed for irregular menstruation, where the subgroup difference was statistically significant and the I² value for Vietnam was 0%. However, this finding should be interpreted cautiously because of the limited number of studies. Overall, these results suggest that geographic location alone does not fully explain the observed variability. Differences in case definitions, measurement tools, survey timing, such as before versus during the COVID-19 pandemic, and participant characteristics likely contributed to this heterogeneity. These findings highlight important limitations in the current literature on menstrual disturbances among medical students. Therefore, the prevalence estimates reported in this review should be viewed less as exact numerical values and more as evidence of a consistent pattern. Despite substantial heterogeneity, menstrual disturbances repeatedly appeared as a common health burden among female medical students in Southeast Asia, and several related factors were reported across studies.

#### Prevalence in relation to existing evidence

The prevalence of dysmenorrhea in this review was higher than that commonly reported in population-based studies of reproductive-aged women, but it was comparable to estimates reported among university and health science students in other regions. Among women of reproductive age in the general population, previous global meta-analyses reported dysmenorrhea prevalence estimates of 71.3%, and a study in Iran reported the percentage of 73.27%, compared with 78.11% among female medical students in this review [3, 33]. Compared with estimates from other continents, the prevalence in Southeast Asia was relatively high at 78.11%, exceeding the range of 59.4% to 72.2% reported in most regions, although it remained lower than the estimate reported for Central America at 89.6% [3]. This finding may reflect the continuing burden of menstrual poverty and infrastructural barriers faced by women in Southeast Asia compared with those in other regions.

#### Associated factors and plausible pathways

Among the correlates examined in this review, family history showed a consistent positive association with menstrual disturbances. Most studies reported statistically significant associations, with odds ratios ranging from 1.47 to 3.76. This pattern supports a plausible hereditary component, consistent with the recognized polygenic nature of menstrual pain and cycle dysregulation [34]. Several studies also reported associations between different types of menstrual disturbances. In particular, PMS was the outcome most frequently reported to co-occur with other menstrual disturbances, including dysmenorrhea, heavy menstrual bleeding, and abnormal menstrual cycle length. This clustering of symptoms may increase the overall burden among female students and highlights the need for effective management of menstrual disturbances in general, and PMS in particular, among medical students.

Psychological factors, including stress, depression, and impaired concentration, were reported in many studies. Dysmenorrhea was the most frequently affected outcome, which is important because it was also the most commonly reported menstrual disturbance among female medical students. These associations are particularly meaningful in the context of medical education, where sustained concentration and consistent attendance are critical to clinical training outcomes [35]. Importantly, the relationship between psychological distress and menstrual symptoms is likely bidirectional. Menstrual symptoms may worsen psychological burden, while pre-existing psychological distress may amplify symptom perception and severity. Future longitudinal studies are needed to clarify this temporal relationship.

#### Heterogeneity and quality of the evidence

High heterogeneity was observed across studies reporting the effects of lifestyle factors on menstrual disturbances. However, several relatively consistent findings were identified. Caffeine consumption, long working hours, limited rest, and low or irregular physical activity were frequently reported to be associated with adverse menstrual health outcomes. Notably, these factors are common among medical students because of their heavy academic workload and demanding training environment. Therefore, lifestyle-related contributors should be considered when designing interventions to improve menstrual health in this population. Although meta-regression to systematically explore sources of heterogeneity (such as country, year of publication, sample size, or diagnostic criteria) was not feasible in this review due to the small number of studies per outcome (k ≤ 9), these approaches should be pursued in future syntheses as the evidence base grows.

### Sociocultural context and regional heterogeneity

4 countries contributing data to this review, Vietnam, Thailand, Indonesia, and Malaysia, differ substantially in terms of their reproductive healthcare systems, cultural attitudes toward menstruation, and the structure of medical education, all of which may partially explain the variability in reported prevalence. In Vietnam and Indonesia, menstrual health services are often integrated into primary care, but access can be limited in rural or resource-constrained settings, and menstruation remains a taboo subject that discourages help-seeking behavior. In Thailand, students benefit from a comparatively well-developed public health infrastructure, yet high academic pressure in medical programs may sustain elevated menstrual burden. In Malaysia, the multicultural composition of the student population with Malay, Chinese, and Indian students having distinct health beliefs and dietary patterns, may contribute to heterogeneity in menstrual outcomes, as suggested by the significantly elevated odds of dysmenorrhea among Malay students in one included study. Such sociocultural and systemic differences may influence not only the true burden of menstrual disturbances but also the willingness of students to report symptoms. Comparative findings from South Asian populations, where similarly high psychosocial burdens have been reported among women in the perinatal period with prevalence of postpartum depression reaching approximately 28% and significant associations with psychosocial risk factors including low social support and poor marital relationship suggest shared regional patterns in how reproductive health outcomes are shaped by sociocultural context and healthcare access [36]. Future research should systematically account for these country-level contextual factors when designing studies and interpreting cross-national comparisons of menstrual health outcomes in Southeast Asia.

### Strengths and limitations

This review has several strengths that support the quality and transparency of its findings. The protocol was prospectively registered in PROSPERO, which helped ensure methodological transparency and reduce the risk of selective reporting. The review also addresses an important evidence gap regarding menstrual disturbances among female medical students in Southeast Asia, a region with distinctive social, cultural, and infrastructural characteristics that remain underexplored in the literature. In addition, this study examined multiple types of menstrual disturbances, providing a more comprehensive perspective than previous reviews. However, several limitations should be acknowledged. Most included studies used cross-sectional designs, which limits causal inference. In addition, most studies relied on convenience or snowball sampling, increasing the risk of selection bias and reducing representativeness. Standardized definitions for menstrual disturbance outcomes were not consistently used, contributing to substantial heterogeneity across studies. For associated factors, narrative synthesis was performed instead of meta-analysis. Furthermore, publication bias was assessed visually with funnel plots rather than quantitatively with Egger’s test due to the limited number of studies for each outcome. Therefore, the assessment of publication bias may not be fully reliable. In addition, quality appraisal using the JBI checklist showed that nearly half of the included studies, 8 out of 17, were classified as having a high risk of bias, with JBI scores of 3 or lower. The GRADE assessment indicated very low certainty for the pooled prevalence estimates and generally low certainty for the evidence on associated factors. The very low certainty of the evidence across all pooled outcomes further underscores the exploratory nature of these findings, limits the confidence with which any specific prevalence figure can be recommended for policy use, and highlights the critical need for future studies that employ standardized outcome definitions and rigorous designs. Regarding the influence of study quality on pooled estimates, the sensitivity analysis excluding high-risk-of-bias studies for dysmenorrhea yielded a pooled prevalence of 78.09% (95% CI, 62.05%–88.60%), comparable to the primary estimate of 78.11%, with substantial heterogeneity remaining (I² = 98.4%). This suggests that the pooled findings were not substantially driven by lower-quality studies, although similar sensitivity analyses could not be conducted for other outcomes due to an insufficient number of remaining studies after exclusion. Accordingly, the findings of this review should not be interpreted as confirmatory evidence, but rather as exploratory and hypothesis-generating evidence.

## Implications for future research and medical training settings

Based on these findings, future studies should standardize the definitions and measurement tools used to assess menstrual disturbances among students. Longitudinal studies are also needed to clarify the temporal and potential causal relationships between associated factors and specific types of menstrual disturbances. In addition, research should be expanded across Southeast Asia, as the current review identified evidence from only four of the eleven countries in the region.

## Conclusion

Menstrual disturbances were commonly reported among female medical students in Southeast Asia. Family history showed the most consistent positive association, while psychological distress, functional impairment, caffeine intake, limited rest, and low or irregular physical activity were also reported as potential correlates. However, because the evidence was based predominantly on cross-sectional observational studies with heterogeneous definitions, measurement tools, and adjustment strategies, these associations should not be interpreted as causal. Furthermore, data were available from only four of the eleven countries in Southeast Asia (Vietnam, Thailand, Indonesia, and Malaysia), limiting the generalizability of these findings to the broader region. Future research using standardized outcome definitions, validated instruments, and longitudinal designs is needed to clarify the temporal relationships between these factors and specific menstrual disturbance outcomes.

## Supplementary Information

Additional file 1. Search strategies.

Additional file 2. Quality assessment of the included studies using the Joanna Briggs Institute checklist for analytical cross-sectional studies.

Additional file 3. Certainty of evidence assessment for the pooled prevalence outcomes and narrative synthesis of associated factors using the GRADE approach.

Additional file 4. Leave-one-out sensitivity analysis evaluating the influence of individual studies on the pooled prevalence estimates.

Additional file 5. Sensitivity analysis excluding lower-quality studies.

Additional file 6. Publication bias assessment for dysmenorrhea.

Additional file 7. Publication bias assessment for irregular menstruation.

Additional file 8. Publication bias assessment for premenstrual disorders.

Additional file 9. Publication bias assessment for abnormal menstrual cycle duration.

Additional file 10. Structured narrative synthesis of factors associated with menstrual disturbances among female medical students in Southeast Asia.

Additional file 11. List of excluded full-text reports with specific reasons for exclusion.

Additional file 12. PRISMA 2020 checklist.

Additional file 13. Data extraction form.

Additional file 14. Comparison of original PROSPERO protocol criteria and revised criteria with justification for each modification.

## Data Availability

All the data extracted and analyzed during this systematic review and meta-analysis are included in this published article and its supplementary materials.

## Abbreviations

MD: Menstrual Disturbances
PMS: Premenstrual Syndrome
PMDD: Premenstrual Dysphoric Disorder
PMD: Premenstrual disorders
LMICs: Low- and Middle-Income Countries
SEA: Southeast Asia
AMCD: Abnormal Menstrual Cycle Duration
HMB: Heavy Menstrual Bleeding
CI: Confidence Interval
I²: I-squared statistic
PR: Prevalence Ratio
OR: Odds Ratio
PROSPERO: International Prospective Register of Systematic Reviews
PRISMA: Preferred Reporting Items for Systematic Reviews and Meta-Analyses
JBI: Joanna Briggs Institute
COVID-19: Coronavirus Disease 2019
BMI: Body Mass Index
QoL: Quality of Life
Q test: Cochran’s Q heterogeneity test
N: Sample size
Y1-Y6: Academic year levels (Year 1 to Year 6 undergraduate medical students)
