# Supplementary material for: Prevalence of menstrual disturbances and associated factors among female medical students in Southeast Asia: a systematic review and meta-analysis
Source: BMC Womens Health. 2026 Jul 3;26:474. doi: 10.1186/s12905-026-04642-5 (PMC13602656; doi:10.1186/s12905-026-04642-5)
Supplement: Supplementary file 1 [file 12905_2026_4642_MOESM10_ESM.docx]

### **Supplementary Table S1.**

*Associated factors with specific types of menstrual disturbances*

| **Study_ID** | **Author (Year)** | **Factor Category** | **Specific Factor** | **Specific type of MD** | **Effect Measure** | **95% CI** | **p-value** | **JBI** |
| --- | --- | --- | --- | --- | --- | --- | --- | --- |
| 6 | Situmorang (2024) | Family History | Family history of dysmenorrhoea | Primary dysmenorrhoea occurrence | aOR 3.76 | 1.89; 7.45 | <0.001 | 8 |
| 6 | Situmorang (2024) | Family History | Family history of dysmenorrhoea | Primary dysmenorrhoea severity | aOR 1.47 | 1.04; 2.06 | 0.028 | 8 |
| 10 | Do (2022) | Family History | Family history of menstrual disorders | Dysmenorrhea | OR 2.786 | 1.809; 4.290 | <0.001 | 2 |
| 2 | Jaiprakash (2016) | Family History | Family history of dysmenorrhea | Dysmenorrhea | OR 2.69 | 1.37; 5.25 | <0.05 | 5 |
| 7 | Tanmahasamut (2012) | Family History | Family history of dysmenorrhea | Dysmenorrhea | NR | NR | <0.001 | 4 |
| 15 | Tung (2024) | Family History | Family history of menstrual disorders | Menstrual disorders | OR 1.8824 | 1.0770; 3.3318 | 0.0182 | 2 |
| 8 | Ho (2024) | Demographic and academic | Age | Dysmenorrhea | OR 0.77 | 0.44; 1.36 | 0.367 | 8 |
| 11 | Ngo (2023) | Demographic and academic | Age | Premenstrual Syndrome or Premenstrual Dysphoric Disorder | NR | NR | 0.396 | 8 |
| 11 | Ngo (2023) | Demographic and academic | Study year [Clinic (> 3 years)] | Premenstrual Syndrome or Premenstrual Dysphoric Disorder | NR | NR | 0.594 | 8 |
| 6 | Situmorang (2024) | Demographic and academic | Academic level [Clinical] | Primary dysmenorrhoea severity | aOR 1.66 | 0.89; 3.06 | 0.110 | 8 |
| 6 | Situmorang (2024) | Demographic and academic | Age | Primary dysmenorrhoea occurrence | aOR 1.20 | 0.96; 1.49 | 0.102 | 8 |
| 6 | Situmorang (2024) | Demographic and academic | Age | Primary dysmenorrhoea severity | aOR 0.99 | 0.82; 1.20 | 0.924 | 8 |
| 6 | Situmorang (2024) | Demographic and academic | Indonesian of foreign descent | Primary dysmenorrhoea severity | aOR 0.50 | 0.30; 0.83 | 0.008 | 8 |
| 12 | Tran (2023) | Demographic and academic | Bachelor’s program | PMS | aOR 1.81 | 1.1; 2.9 | 0.023 | 8 |
| 2 | Jaiprakash (2016) | Demographic and academic | Race: Malay | Dysmenorrhoea | aOR 5.70 | 1.13; 28.78 | <0.05 | 5 |
| 13 | Le (2024) | Demographic and academic | Major (General medicine) | PMS | aOR 2.38 | 1.15; 4.96 | 0.02 | 1 |
| 7 | Tanmahasamut (2012) | Demographic and academic | Age | Dysmenorrhea | NR | NR | 0.065 | 4 |
| 4 | Tran (2025) | Demographic and academic | Academic year | Menstrual change | OR 1.248 | 1.118; 1.392 |  | 4 |
| 4 | Tran (2025) | Demographic and academic | Age | Menstrual disturbances | OR 1.114 | 1.057; 1.175 |  | 4 |
| 4 | Tran (2025) | Demographic and academic | Major (Pharmacy vs. Medicine) | Menstrual change | OR 0.805 | 0.692; 0.937 |  | 4 |
| 15 | Tung (2024) | Demographic and academic | Residence before university admission: rural | Menstrual disoders | 1.8628 | 1.1228; 3.0923 | 0.0104 | 2 |
| 33 | Joseph (2013) | Psychological and Quality of life | HRQoL mental component summary score | Dysmenorrhea | Adjusted mean difference -3.4 | NR | <0.001 | 8 |
| 33 | Joseph (2013) | Psychological and Quality of life | HRQoL physical component summary score | Dysmenorrhea | Adjusted mean difference -4.9 | NR | <0.001 | 8 |
| 9 | Situmorang (2025) | Psychological and Quality of life | Absenteeism | Dysmenorrhoea occurrence | aOR 12.10 | 1.65; 88.83 | 0.014 | 6 |
| 9 | Situmorang (2025) | Psychological and Quality of life | Absenteeism | Dysmenorrhoea severity | aOR 5.65 | 3.32; 9.63 | <0.001 | 6 |
| 9 | Situmorang (2025) | Psychological and Quality of life | Activity disruption | Dysmenorrhoea occurrence | aOR 34.95 | 4.77; 256.16 | <0.001 | 6 |
| 9 | Situmorang (2025) | Psychological and Quality of life | Activity disruption | Dysmenorrhoea severity | aOR 6.92 | 4.63; 10.36 | <0.001 | 6 |
| 9 | Situmorang (2025) | Psychological and Quality of life | Concentration disruption | Dysmenorrhoea occurrence | aOR 12.92 | 6.14; 27.22 | <0.001 | 6 |
| 9 | Situmorang (2025) | Psychological and Quality of life | Concentration disruption | Dysmenorrhoea severity | aOR 7.24 | 4.68; 11.19 | <0.001 | 6 |
| 9 | Situmorang (2025) | Psychological and Quality of life | Depression occurrence | Dysmenorrhoea occurrence | aOR 2.16 | 1.23; 3.81 | 0.007 | 6 |
| 9 | Situmorang (2025) | Psychological and Quality of life | Depression occurrence | Dysmenorrhoea severity | aOR 2.07 | 1.47; 2.92 | <0.001 | 6 |
| 9 | Situmorang (2025) | Psychological and Quality of life | Quality of life | Dysmenorrhoea occurrence | aOR -1.82 | -2.63; 1.02 | <0.001 | 6 |
| 9 | Situmorang (2025) | Psychological and Quality of life | Stress occurence | Dysmenorrhoea severity | aOR 1.82 | 1.26; 2.62 | <0.001 | 6 |
| 2 | Jaiprakash (2016) | Psychological and Quality of life | Interference with social life | Dysmenorrhoea | OR 2.00 | 0.94; 1.72 | <0.05 | 5 |
| 5 | Pham (2024) | Psychological and Quality of life | Affected quality of life | Dysmenorrhea | NR | NR | 0.039 | 1 |
| 5 | Pham (2024) | Psychological and Quality of life | Stress rating | Irregular menstrual cycle | NR | NR | 0.007 | 1 |
| 3 | Soe (2017) | Psychological and Quality of life | Daily routine activities | Dysmenorrhoea | aOR 2.68 | 1.60; 4.49 | <0.001 | 2 |
| 3 | Soe (2017) | Psychological and Quality of life | Daily routine activities | PMS | OR 2.47 | 1.26; 4.83 | 0.008 | 2 |
| 1 | Taufiq (2019) | Psychological and Quality of life | Stress level | Abnormal menstrual cycle | POR 1.977 | 1.273; 3.068 | 0.002 | 2 |
| 11 | Ngo (2023) | Dietary Habits and lifestyle | Alcohol consumption | PMS/PMDD | NR | NR | 0.523 | 8 |
| 11 | Ngo (2023) | Dietary Habits and lifestyle | Consuming caffeine more than four times per week | Premenstrual Syndrome or Premenstrual Dysphoric Disorder | PR 2.86 | 1.24; 6.58 | 0.014 | 8 |
| 11 | Ngo (2023) | Dietary Habits and lifestyle | Physical activity | MPS/MPDD Moderate Vigorous | NR | NR | 0.158 0.267 | 8 |
| 12 | Tran (2023) | Dietary Habits and lifestyle | Exercising | PMS | aOR 2.4 | 0.9; 5.8 | 0.062 | 8 |
| 13 | Le (2024) | Dietary Habits and lifestyle | Alcohol consumption | PMS | aOR 1.58 | 0,71; 3,54 | 0.266 | 1 |
| 13 | Le (2024) | Dietary Habits and lifestyle | Coffee consumption | PMS | aOR 3.10 | 1.21; 7.95 | 0.019 | 1 |
| 13 | Le (2024) | Dietary Habits and lifestyle | Eating fruits/vegetables | PMS | aOR 2.92 | 1,41; 6,07 | 0.004 | 1 |
| 13 | Le (2024) | Dietary Habits and lifestyle | Electronics use (more than 6 hours/day) | PMS | aOR 2.40 | 1.19; 4.87 | 0.015 | 1 |
| 13 | Le (2024) | Dietary Habits and lifestyle | Excersing (<150 minutes/week) | PMS | aOR 6.70 | 1.36; 32.96 | 0.019 | 1 |
| 13 | Le (2024) | Dietary Habits and lifestyle | Sleeping hours (< 7 hours/day) | PMS | aOR 2.46 | 1,25; 4,84 | 0.009 | 1 |
| 13 | Le (2024) | Dietary Habits and lifestyle | Tea consumption | PMS | aOR 2.31 | 1.04; 5.16 | 0.041 | 1 |
| 13 | Le (2024) | Dietary Habits and lifestyle | Working/Studying hours (more than 8 hours/day) | PMS | aOR 3.28 | 1.60; 6.71 | 0.001 | 1 |
| 5 | Pham (2024) | Dietary Habits and lifestyle | Physical activity | Dysmenorrhea | NR | NR | 0.038 | 1 |
| 3 | Soe (2017) | Dietary Habits and lifestyle | Eating fast food | Dysmenorrhea | OR 0.77 | 0.47; 1.27 | 0.31 | 2 |
| 3 | Soe (2017) | Dietary Habits and lifestyle | Eating fast food | PMS | OR 2.25 | 1.14; 4.45 | 0.02 | 2 |
| 3 | Soe (2017) | Dietary Habits and lifestyle | Regular exercise | Dysmenorrhea | OR 1.07 | 0.65; 1.76 | 0.79 | 2 |
| 3 | Soe (2017) | Dietary Habits and lifestyle | Regular exercise | PMS | OR 0.79 | 0.41; 1.49 | 0.46 | 2 |
| 7 | Tanmahasamut (2012) | Dietary Habits and lifestyle | Alcohol consumption | Dysmenorrhea | NR | NR | 0.116 | 4 |
| 7 | Tanmahasamut (2012) | Dietary Habits and lifestyle | Coffee consumption | Dysmenorrhea | NR | NR | 0.743 | 4 |
| 7 | Tanmahasamut (2012) | Dietary Habits and lifestyle | Exercising | Dysmenorrhea | NR | NR | 0.235 | 4 |
| 4 | Tran (2025) | Dietary Habits and lifestyle | Bedtime | Menstrual change | OR 1.064 | 1.011; 1.120 | NR | 4 |
| 4 | Tran (2025) | Dietary Habits and lifestyle | Clinic consultation (yes) | Menstrual change | OR 1.183 | 1.059; 1.321 |  | 4 |
| 4 | Tran (2025) | Dietary Habits and lifestyle | Electronics use (h/day) | Menstrual change | OR 1.096 | 1.051; 1.144 |  | 4 |
| 4 | Tran (2025) | Dietary Habits and lifestyle | PMS knowledge (since school) | Menstrual change | OR 0.848 | 0.759; 0.946 |  | 4 |
| 4 | Tran (2025) | Dietary Habits and lifestyle | Sleep hours | Menstrual change | OR 0.976 | 0.914; 1.043 |  | 4 |
| 15 | Tung (2024) | Dietary Habits and lifestyle | Sleeping hours (less than 5 hours/day) (6-8 hours/day) | Menstrual disoders | OR 1.5152 OR 1.1033 | 0.4292; 5.3233 0.4044; 2.9728 | 0.4646 0.8286 | 2 |
| 15 | Tung (2024) | Dietary Habits and lifestyle | Sleeping time After 12AM | Menstrual disoders | OR 1.0657 | 0.4882; 2.2969 | 0.8605 | 2 |
| 11 | Ngo (2023) | Reproductive and medical history | Having negative Rh blood type | PMS/PMDD | PR 4.43 | 1.95; 10.08 | <0.001 | 8 |
| 11 | Ngo (2023) | Reproductive and medical history | Number of menstrual days | PMS/PMDD | PR 0.958 | 0.835; 1.101 | 0.548 | 8 |
| 12 | Tran (2023) | Reproductive and medical history | Dysmonerrhea | PMS | OR 14.6 | 2.9; 73.2 | 0.001 | 8 |
| 12 | Tran (2023) | Reproductive and medical history | Heavy menstrual bleeding | PMS | OR 5.4 | 1.3; 24.7 | 0.031 | 8 |
| 12 | Tran (2023) | Reproductive and medical history | Heavy menstrual bleeding | PMS | aOR 25.7 | 2.5; 265.3 | 0.005 | 8 |
| 14 | Hung (2024) | Reproductive and medical history | Menstrual Duration 1-3 days 4-7 days Over 7 days | PMS severity | aOR 0.75 aOR 0.56 1.0 | 0.12; 4.63 0.11; 2.92 | 0.75 0.49 | 3 |
| 14 | Hung (2024) | Reproductive and medical history | Menstrual pain level Moderate Severe | PMS severity | aOR 3.81 aOR 10.04 | 1.18; 12.31 2.59; 38.92 | 0.02 <0.01 | 3 |
| 13 | Le (2024) | Reproductive and medical history | Abnormal length | PMS | aOR 2.08 | 0.83; 5.23 | 0.12 | 1 |
| 13 | Le (2024) | Reproductive and medical history | Dysmonerrhea | PMS | aOR 4.06 | 1.82; 9.07 | 0.001 | 1 |
| 4 | Tran (2025) | Reproductive and medical history | Blood group A | Menstrual change | OR 1.137 | 1.029; 1.256 | NR | 4 |
| 4 | Tran (2025) | Reproductive and medical history | Blood group B | Menstrual change | OR 1.124 | 1.034; 1.222 | NR | 4 |
| 4 | Tran (2025) | Reproductive and medical history | Rh factor | Menstrual change | OR 1.078 | 1.000; 1.162 | NR | 4 |
| 16 | Vu (2025) | Reproductive and medical history | Abdominal pain before menstruation | Dysmenorrhea | NR | NR | <0.001 | 2 |
| Note. NR = Not reported; HRQoL = health-related quality of life; PCS = physical component summary; MCS = mental component summary; MD = menstrual disturbance. | | | | | | | | |
