# Supplementary material for: Prevalence of menstrual disturbances and associated factors among female medical students in Southeast Asia: a systematic review and meta-analysis
Source: BMC Womens Health. 2026 Jul 3;26:474. doi: 10.1186/s12905-026-04642-5 (PMC13602656; doi:10.1186/s12905-026-04642-5)
Supplement: Supplementary file 3 [file 12905_2026_4642_MOESM14_ESM.docx]

**Table.** Comparison of original PROSPERO protocol criteria and revised criteria

| **Protocol Criterion** | **Original PROSPERO Protocol** | **Revised Criteria Applied in This Review** |
| --- | --- | --- |
| **Review title** | Prevalence of menstrual disturbances, associated factors, and menstrual hygiene barriers among female medical students in Southeast Asia: A systematic review and meta-analysis | Prevalence of menstrual disturbances and associated factors among female medical students in Southeast Asia: A systematic review and meta-analysis |
| **Primary outcomes** | (1) Pooled prevalence of overall menstrual disturbances and specific subtypes: dysmenorrhea, irregular menstrual cycles, PMS/PMDD, Heavy Menstrual Bleeding (HMB) (2) Pooled effect estimates (ORs/RRs) for associated factors (academic stress, year of study, sleep quality, BMI) | (1) Pooled prevalence of four operationally measurable subtypes: dysmenorrhea, irregular menstruation, PMS/PMDD, and abnormal menstrual cycle duration (AMCD) (2) Associated factors reported as narrative synthesis only (no pooled ORs/RRs) |
| **Additional outcomes (Objectives 3 and 4)** | Obj 3: Prevalence/narrative synthesis of menstrual hygiene barriers (MHB) and period poverty  Obj 4: Academic impact (absenteeism, concentration) Also: PMS/PMDD severity scores; KAP narrative synthesis | Obj 3 and 4 not reported quantitatively; brief narrative retained in Discussion regarding academic impact where reported |
| **Search databases** | CENTRAL (Cochrane), Embase via Ovid, Embase.com, MEDLINE, PubMed, Scopus (6 databases) | PubMed, Embase, Scopus (3 databases) + Google Scholar (first 200 relevance-ranked records) + manual searching and citation tracking |
| **Search end date** | 30 September 2025 | 6 May 2026 |
| **Unpublished studies** | Both published and unpublished studies sought | Published peer-reviewed articles and eligible publicly available gray-literature reports with extractable quantitative data. |
| **Meta-regression** | Planned if sufficient studies available to explore study-level covariates | Not performed |
| **Subgroup analyses** | Planned by: year of study (pre-clinical vs clinical), country/sub-region, type of disturbance, study quality, time period, sampling method | Performed by country for dysmenorrhea, irregular menstruation, and AMCD; other planned subgroups not performed |
| **Sensitivity analyses** | Planned by excluding high-risk-of-bias studies or those using non-standard definitions of MD | Performed for dysmenorrhea only (exclusion of lower-quality studies); could not be performed for other outcomes |

*Abbreviations: PMS, premenstrual syndrome; PMDD, premenstrual dysphoric disorder; HMB, heavy menstrual bleeding; AMCD, abnormal menstrual cycle duration; MHB, menstrual hygiene barriers; KAP, knowledge, attitudes, and practices; JBI, Joanna Briggs Institute; GRADE, Grading of Recommendations, Assessment, Development and Evaluations; OR, odds ratio; RR, risk ratio; SEA, Southeast Asia; k, number of included studies.*
