# Supplementary material for: Prevalence of menstrual disturbances and associated factors among female medical students in Southeast Asia: a systematic review and meta-analysis
Source: BMC Womens Health. 2026 Jul 3;26:474. doi: 10.1186/s12905-026-04642-5 (PMC13602656; doi:10.1186/s12905-026-04642-5)
Supplement: Supplementary file 4 [file 12905_2026_4642_MOESM1_ESM.docx]

| PUBMED | ("Menstruation Disturbances"[Mesh] OR "Dysmenorrhea"[Mesh] OR "Premenstrual Syndrome"[Mesh] OR "menstrual"[tiab] OR "dysmenorrhea"[tiab] OR "premenstrual"[tiab] OR "PMDD"[tiab] OR "PMS"[tiab]) AND ("Students, Medical"[Mesh] OR "Students, Health Occupations"[Mesh] OR "Students, Nursing"[Mesh] OR "medical student*"[tiab] OR "health science student*"[tiab] OR "healthcare student*"[tiab] OR "dental student*"[tiab] OR "nursing student*"[tiab] OR "healthcare professional*"[tiab]) AND (2000:2026[pdat]) | 406 results |
| --- | --- | --- |
| Embase | ('menstruation disorder'/exp OR 'dysmenorrhea'/exp OR 'premenstrual syndrome'/exp OR 'menstrual':ti,ab OR 'dysmenorrhea':ti,ab OR 'premenstrual':ti,ab OR 'pmdd':ti,ab OR 'pms':ti,ab) AND ('medical student'/exp OR 'medical student*':ti,ab OR 'health science student*':ti,ab OR 'healthcare student*':ti,ab OR 'dental student*':ti,ab OR 'nursing student*':ti,ab OR 'healthcare professional*':ti,ab)/lim | 1031 results |
| Scopus | ( TITLE-ABS-KEY ( "menstrual" OR "dysmenorrhea" OR "premenstrual" OR "PMDD" OR "PMS" ) AND TITLE-ABS-KEY ( "medical student*" OR "health science student*" OR "healthcare student*" OR "dental student*" OR "nursing student*" OR "healthcare professional*" ) ) AND PUBYEAR > 1999 | 698 results |
| Google scholar | allintitle: "menstrual" OR "dysmenorrhea" "medical students" |  |
