# Supplementary material for: Prevalence of menstrual disturbances and associated factors among female medical students in Southeast Asia: a systematic review and meta-analysis
Source: BMC Womens Health. 2026 Jul 3;26:474. doi: 10.1186/s12905-026-04642-5 (PMC13602656; doi:10.1186/s12905-026-04642-5)
Supplement: Supplementary file 5 [file 12905_2026_4642_MOESM3_ESM.docx]

**Additional file 3. GRADE Certainty Assessment and Summary of Findings**

# Worksheet 1. Relative importance of outcomes selected for the Summary of Findings table

Ratings follow the EPOC 1-9 scale: 1-3 = not important; 4-6 = important but not critical; 7-9 = critical for decision-making.

| **Outcome** | **Reason for inclusion** | **Consensus relative importance** | **Included in SoF table** |
| --- | --- | --- | --- |
| Dysmenorrhea | Primary or major symptom-specific prevalence outcome | 9 (critical) | Yes |
| Irregular menstruation | Primary or major symptom-specific prevalence outcome | 8 (critical) | Yes |
| Premenstrual disorders (PMS/PMDD) | Primary or major symptom-specific prevalence outcome | 8 (critical) | Yes |
| Abnormal menstrual cycle duration (AMCD) | Primary or major symptom-specific prevalence outcome | 7 (critical) | Yes |
| Overall menstrual disorders | Primary or major symptom-specific prevalence outcome | 7 (critical) | Yes |
| Heavy menstrual bleeding (HMB) | Primary or major symptom-specific prevalence outcome | 7 (critical) | Yes |
| Menstrual change | Reported outcome included for completeness, but not critical for decision-making | 6 (important) | Yes |

# Worksheet 2. Certainty assessment of evidence across studies for each outcome

Certainty ratings are outcome-specific. Ratings reached the GRADE floor of very low for all prevalence outcomes; additional downgrading beyond this floor is shown in the domain-level comments but does not reduce the final category further.

| **Outcome** | **No. of studies** | **Design** | **Risk of bias** | **Inconsistency** | **Indirectness** | **Imprecision** | **Other considerations** | **Certainty (overall)** |
| --- | --- | --- | --- | --- | --- | --- | --- | --- |
| Dysmenorrhea | 9 | Cross-sectional studies (non-randomised evidence; initial certainty: low) | Serious (-1) | Very serious (-2) | No serious | Serious (-1) | Publication bias unclear; no upgrading | ⊕○○○ Very low |
| Irregular menstruation | 6 | Cross-sectional studies (non-randomised evidence; initial certainty: low) | Serious (-1) | Very serious (-2) | No serious to serious | Serious (-1) | Publication bias unclear; no upgrading | ⊕○○○ Very low |
| Premenstrual disorders (PMS/PMDD) | 5 | Cross-sectional studies (non-randomised evidence; initial certainty: low) | Serious (-1) | Very serious (-2) | Serious (-1) | Serious (-1) | Publication bias unclear; no upgrading | ⊕○○○ Very low |
| Abnormal menstrual cycle duration (AMCD) | 5 | Cross-sectional studies (non-randomised evidence; initial certainty: low) | Serious (-1) | Very serious (-2) | Serious (-1) | Serious (-1) | Publication bias unclear; no upgrading | ⊕○○○ Very low |
| Overall menstrual disorders | 3 | Cross-sectional studies (non-randomised evidence; initial certainty: low) | Serious (-1) | Very serious (-2) | Serious (-1) | Serious (-1) | Publication bias not estimable; no upgrading | ⊕○○○ Very low |
| Heavy menstrual bleeding (HMB) | 3 | Cross-sectional studies (non-randomised evidence; initial certainty: low) | Serious (-1) | Very serious (-2) | Serious (-1) | Serious (-1) | Publication bias not estimable; no upgrading | ⊕○○○ Very low |
| Menstrual change | 1 | Single cross-sectional study (non-randomised evidence; initial certainty: low) | Serious (-1) | Not estimable | Serious (-1) | Very serious (-2) | Publication bias not estimable; no upgrading | ⊕○○○ Very low |

# Worksheet 3. Summary of Findings table

**Summary of findings: prevalence of menstrual disturbances among female medical students in Southeast Asia**

People: female undergraduate medical students. Settings: Southeast Asian countries represented in the included studies. Evidence base: 17 cross-sectional studies including 7,080 participants overall. Synthesis: random-effects meta-analysis when sufficient comparable prevalence data were available; structured narrative synthesis otherwise.

| **Outcome** | **Summary estimate / impact** | **Participants (studies)** | **Certainty of evidence (GRADE)** | **Main reasons for downgrading** | **Interpretation** |
| --- | --- | --- | --- | --- | --- |
| Dysmenorrhea | 78.11% (95% CI, 67.07-86.21); prediction interval, 31.24-96.56; I2 = 97.9% | 3,950 participants (9 studies) | ⊕○○○ Very low | Downgraded for serious risk of bias, very serious unexplained heterogeneity, and serious imprecision reflected by a very wide prediction interval. | Available studies suggest dysmenorrhea may be common, but the exact prevalence is uncertain because the certainty of evidence is very low. |
| Irregular menstruation | 30.10% (95% CI, 18.54-44.89); prediction interval, 4.62-79.29; I2 = 98.0% | 2,621 participants (6 studies) | ⊕○○○ Very low | Downgraded for serious risk of bias, very serious inconsistency, and serious imprecision. Indirectness was considered borderline because case definitions varied across studies. | Available studies suggest irregular menstruation occurs in a substantial minority of students, but the exact prevalence is uncertain because the certainty of evidence is very low. |
| Premenstrual disorders (PMS/PMDD) | 39.60% (95% CI, 20.13-63.05); prediction interval, 2.37-94.65; I2 = 98.5% | 1,597 participants (5 studies) | ⊕○○○ Very low | Downgraded for serious risk of bias, very serious inconsistency, serious indirectness from combined PMS/PMDD definitions and differing measurement tools, and serious imprecision. | Available studies suggest premenstrual disorders may be frequent, but the exact prevalence is uncertain because the certainty of evidence is very low. |
| Abnormal menstrual cycle duration (AMCD) | 22.58% (95% CI, 15.48-31.72); prediction interval, 5.73-58.34; I2 = 95.0% | 2,227 participants (5 studies) | ⊕○○○ Very low | Downgraded for serious risk of bias, very serious inconsistency, serious indirectness due to variable operational definitions of abnormal cycle duration, and serious imprecision. | Available studies suggest AMCD may affect a minority of students, but the exact prevalence is uncertain because the certainty of evidence is very low. |
| Overall menstrual disorders | Not pooled. Individual estimates: 22.3%, 39.7%, and 55.5% | 941 participants (3 studies) | ⊕○○○ Very low | Downgraded for serious methodological limitations, substantial variability in estimates, indirectness from broad and non-standardised outcome definitions, and limited information from only three studies. | It is uncertain how common overall menstrual disorders are because the certainty of evidence is very low and the outcome was defined inconsistently. |
| Heavy menstrual bleeding (HMB) | Not pooled. Individual estimates: 19.4%, 27.4%, and 57.0% | 1,094 participants (3 studies) | ⊕○○○ Very low | Downgraded for serious risk of bias, inconsistent estimates, probable measurement differences, and limited information from only three studies. | It is uncertain how common heavy menstrual bleeding is because the certainty of evidence is very low. |
| Menstrual change | 49.8% in one study | 884 participants (1 studies) | ⊕○○○ Very low | Downgraded for single-study evidence, risk of bias, uncertain applicability, and very serious imprecision for decision-making across Southeast Asia. | It is uncertain how common menstrual change is because evidence comes from one study and the certainty of evidence is very low. |

# Summary of findings: Narrative certainty assessment for associated factors

A meta-analysis of associated factors was not performed because studies were substantially heterogeneous in effect measures, model specifications, and adjustment strategies. Certainty was therefore summarised narratively and should not be interpreted as evidence of causality. Associated factors were not meta-analysed because the included studies differed substantially in effect measures, model specifications, exposure definitions, and adjustment strategies. Certainty was therefore assessed narratively by factor domain. Because all contributing studies were cross-sectional, the starting certainty was low; ratings were downgraded when there were serious concerns about bias, inconsistency, indirectness, imprecision, or publication bias. These findings should not be interpreted as causal evidence.

| **Associated factor domain** | **Summary of evidence from higher-priority and supporting studies** | **Certainty** | **Judgement** |
| --- | --- | --- | --- |
| **Family history** | The direction of evidence was consistently positive. Higher-priority studies reported associations between family history of dysmenorrhoea and primary dysmenorrhoea occurrence (aOR = 3.76, 95% CI: 1.89-7.45) and severity (aOR = 1.47, 95% CI: 1.04-2.06), and between family history and dysmenorrhoea (OR = 2.69, 95% CI: 1.37-5.25). Supporting lower-priority studies also reported positive associations with dysmenorrhoea or menstrual disorders, including ORs of 2.786 and 1.8824, and one study reporting p < .001 without an effect estimate. | Low to very low | Most consistent domain. Evidence suggests an association with dysmenorrhoea or menstrual disorders, but certainty remains limited by cross-sectional design, residual confounding, non-standardised outcome definitions, and low-quality supporting studies. |
| **Demographic and academic factors** | Age and academic level were mostly not associated with menstrual outcomes in higher-priority studies. Reported null findings included age for dysmenorrhoea or PMS/PMDD and clinical academic level or study year. Isolated associations were reported for Indonesian students of foreign descent and lower dysmenorrhoea severity (aOR = 0.50, 95% CI: 0.30-0.83), bachelor program and PMS (aOR = 1.81, 95% CI: 1.1-2.9), and Malay ethnicity and dysmenorrhoea (aOR = 5.70, 95% CI: 1.13-28.78). Several lower-priority studies reported additional context-specific associations with age, academic year, major, or residence. | Very low | Evidence is inconsistent and context-specific. Age and academic level were generally null in higher-priority studies; other signals were isolated, often imprecise, and not directly comparable across countries or institutions. |
| **Reproductive and medical history / co-occurring menstrual disturbances** | Higher-priority studies suggested clustering of menstrual symptoms. Dysmenorrhoea was associated with PMS (OR = 14.6, 95% CI: 2.9-73.2), and heavy menstrual bleeding was associated with PMS in crude and adjusted analyses (OR = 5.4, 95% CI: 1.3-24.7; aOR = 25.7, 95% CI: 2.5-265.3). Negative Rh blood type was associated with PMS/PMDD (PR = 4.43, 95% CI: 1.95-10.08), whereas number of menstrual days was not clearly associated. Lower-priority studies reported associations involving menstrual pain level, dysmenorrhoea, blood group, Rh factor, abnormal cycle length, and abdominal pain before menstruation. | Very low | Strong estimates were reported, but confidence is very limited. Several predictors were themselves menstrual symptoms or related outcomes, so findings are better interpreted as co-occurrence rather than independent risk factors. Wide confidence intervals and lack of temporality further lower certainty. |
| **Psychological distress, quality of life, and functional impairment** | Higher-priority studies reported associations between dysmenorrhoea and reduced health-related quality of life, psychological distress, and functional impairment. Dysmenorrhoea was associated with lower adjusted physical and mental HRQoL scores (adjusted mean differences: -4.9 and -3.4; both p < .001). Another higher-priority study reported associations with absenteeism (aOR range = 5.65-12.10), activity disruption (aOR range = 6.92-34.95), concentration disruption (aOR range = 7.24-12.92), depression (aOR range = 2.07-2.16), and stress occurrence for dysmenorrhoea severity (aOR = 1.82, 95% CI: 1.26-2.62). Lower-priority studies also reported links with stress, daily routine activities, and quality of life. | Very low | Plausible and clinically relevant, but highly vulnerable to reverse causation and conceptual overlap. Functional impairment may be a consequence of dysmenorrhoea rather than a determinant. Several estimates were large but imprecise. |
| **Dietary habits and lifestyle factors** | Higher-priority evidence was mixed. Frequent caffeine intake was associated with PMS/PMDD (PR = 2.86, 95% CI: 1.24-6.58), whereas alcohol consumption and moderate or vigorous physical activity were not clearly associated. Exercise showed a non-significant estimate for PMS (aOR = 2.40, 95% CI: 0.90-5.80). Lower-priority studies reported additional associations for coffee consumption, fruit/vegetable intake, electronics use, low exercise, short sleep, tea consumption, and long working/studying hours; however, other studies reported null findings for fast food, regular exercise, alcohol, coffee, and sleep-related variables. | Very low | Frequent caffeine intake showed the clearest signal, but overall lifestyle evidence remains very uncertain. Exposure definitions were inconsistent, self-reported, and vulnerable to uncontrolled confounding and selective reporting. |

**Worksheet 4. Plain-language summary statements**

The wording below follows the EPOC recommendation to align plain-language statements with the certainty of evidence. Since all main prevalence outcomes were rated very low certainty, each statement explicitly avoids confirmatory language.

• Available studies suggest dysmenorrhea may be common, but the exact prevalence is uncertain because the certainty of evidence is very low.

• Available studies suggest irregular menstruation occurs in a substantial minority of students, but the exact prevalence is uncertain because the certainty of evidence is very low.

• Available studies suggest premenstrual disorders may be frequent, but the exact prevalence is uncertain because the certainty of evidence is very low.

• Available studies suggest AMCD may affect a minority of students, but the exact prevalence is uncertain because the certainty of evidence is very low.

• It is uncertain how common overall menstrual disorders are because the certainty of evidence is very low and the outcome was defined inconsistently.

• It is uncertain how common heavy menstrual bleeding is because the certainty of evidence is very low.

• It is uncertain how common menstrual change is because evidence comes from one study and the certainty of evidence is very low.

# Footnotes and decision rules

CI = confidence interval; GRADE = Grading of Recommendations Assessment, Development and Evaluation; HMB = heavy menstrual bleeding; PMS = premenstrual syndrome; PMDD = premenstrual dysphoric disorder; SoF = Summary of Findings. aOR = adjusted odds ratio; HRQoL = health-related quality of life; JBI = Joanna Briggs Institute; OR = odds ratio.

All primary studies contributing to the review were cross-sectional; the initial certainty was therefore low for all outcomes.

Risk of bias was judged serious because many included studies used convenience, snowball, purposive, or otherwise non-probability sampling; several studies reported limited information on measurement validity and confounding control; and the manuscript notes that the majority of studies were of low to moderate methodological quality.

Inconsistency was judged very serious for pooled outcomes because heterogeneity was very high across all meta-analyses (I2 = 95.0% to 98.5%), and subgroup analyses generally did not adequately explain the observed variability.

Indirectness was judged serious where outcome definitions were broad, combined, or inconsistent across studies, particularly for PMS/PMDD, AMCD, overall menstrual disorders, HMB, and menstrual change.

Imprecision was judged serious or very serious where 95% CIs and/or prediction intervals were wide, where only a few studies contributed data, or where evidence came from a single study.

Publication bias was not downgraded as a separate domain for the pooled outcomes because Egger’s regression test was not performed due to fewer than 10 studies per outcome, and funnel plots were interpreted as illustrative only. For outcomes with fewer than three or narrative-only studies, publication bias was considered not estimable.

For narrative outcomes, approximate event counts were calculated from the prevalence and sample size reported in the manuscript where exact event counts were not fully specified. These approximations are intended only to describe the amount of information and should not be used as new primary analyses.
